# The impact of empirical Marshall vein ethanol infusion as a first-choice intraoperative strategy on the long-term outcomes in patients with persistent atrial fibrillation undergoing mitral isthmus ablation

**DOI:** 10.3389/fcvm.2023.1223064

**Published:** 2023-08-15

**Authors:** Xianfeng Du, Chenxu Luo, Caijie Shen, Yao Xu, Mingjun Feng, He Jin, Guohua Fu, Binhao Wang, Jin Liu, Fang Gao, Huimin Chu

**Affiliations:** ^1^ Arrhythmia Center, The First Affiliated Hospital of Ningbo University, Ningbo First Hospital, Ningbo, China; ^2^ Key Laboratory of Precision Medicine for Atherosclerotic Diseases of Zhejiang Province, Ningbo, China; ^3^ School of Medicine, Ningbo University, Ningbo, China; ^4^ Department of Neurology, The First Affiliated Hospital of Ningbo University, Ningbo First Hospital, Ningbo City, China

**Keywords:** atrial fibrillation, catheter ablation, mitral isthmus, Marshall vein, ethanol infusion

## Abstract

**Background:**

Marshall vein ethanol infusion (MVEI) as an additional therapy to conventional catheter ablation (CA) has been proved to be efficacious in patients with persistent atrial fibrillation (PeAF). However, whether empirical MVEI could be the first-line strategy in mitral isthmus (MI) ablation has seldom been investigated. Here, we aim to compare the efficacy, safety, and long-term outcomes between provisional and empirical MVEI in PeAF patients undergoing the index MI ablation procedure.

**Methods:**

We enrolled 133 patients with PeAF either in the provisional group (*n* = 38, MVEI was performed when conventional endocardial and/or epicardial ablation procedures were inadequate to achieve bidirectional MI block) or in the empirical group (*n* = 95, MVEI was performed empirically before MI CA).

**Results:**

All of the baseline characteristics were comparable. Less spontaneous or inducible atrial tachycardias (ATs) were encountered in the empirical group of patients (*P *< 0.001). More epicardial ablations were applied (26.3% vs. 9.5%, *P *= 0.016) and a higher incidence of CA-facilitated restoration of sinus rhythm was recorded (86.8% vs. 11.7%, *P *< 0.001) in the provisional group of patients. Although more fluoroscopy time (6.4[4.2, 9.3] vs. 9.5[5.9, 11.6] min, *P *= 0.019) and radiation exposure (69.0[25.3, 160.2] vs. 122.0[62.5, 234.1] mGy, *P *= 0.010) were documented in the empirical group with comparable procedure time, less time (455.9 ± 192.2 vs. 366.5 ± 161.3 s, *P *= 0.038) was consumed to achieve bidirectional MI block during endocardial ablation in the provisional group. Incidences of procedure-related complications were similar between the two groups. During a 16.5 ± 4.4-month follow-up, the empirical group of patients showed a significantly higher rate of freedom from AT recurrence (95.8% vs. 81.6%, log-rank *P *= 0.003), while the rate of freedom from AF or atrial tachyarrhythmias (combining AF and AT) was similar. Both univariate (HR 0.19, 95% CI 0.05–0.64, *P *= 0.008) and multivariate (HR 0.25, 95% CI 0.07–0.92, *P *= 0.037) Cox regression analyses indicated that empirical MVEI was independently associated with lower long-term AT recurrence.

**Conclusion:**

Among patients with PeAF who underwent the index MI ablation procedure, empirical MVEI could reduce endocardial MI ablation time and provide greater long-term freedom from AT recurrence.

## 1. Introduction

Mitral isthmus (MI)-dependent conduction has been considered one of the most important substrates in persistent atrial fibrillation (PeAF) (1). In most cases, bidirectional block of MI could not be achieved only with endocardial catheter ablation (CA), even with supplementary epicardial ablation from the distal coronary sinus (CS). Muscle bundles and vein within the Marshall ligament connecting the left atrium (LA) around the left pulmonary veins (PVs) and CS have been recognized as participants in the maintenance of the AF (2, 3). It is reported that adjunctive Marshall vein ethanol infusion (MVEI) could improve the efficacy of MI ablation and its outcomes in PeAF patients (4–9). However, whether MVEI should be performed empirically before using other conventional ablation strategies or applied provisionally when bidirectional block could not be achieved after conventional ablation steps in PeAF patients undergoing the index MI ablation procedure has been seldom investigated. Against this background, the purpose of this study is to evaluate the efficacy and safety of empirical MVEI in patients with PeAF.

## 2. Materials and methods

### 2.1. Study populations

**Figure 1:**
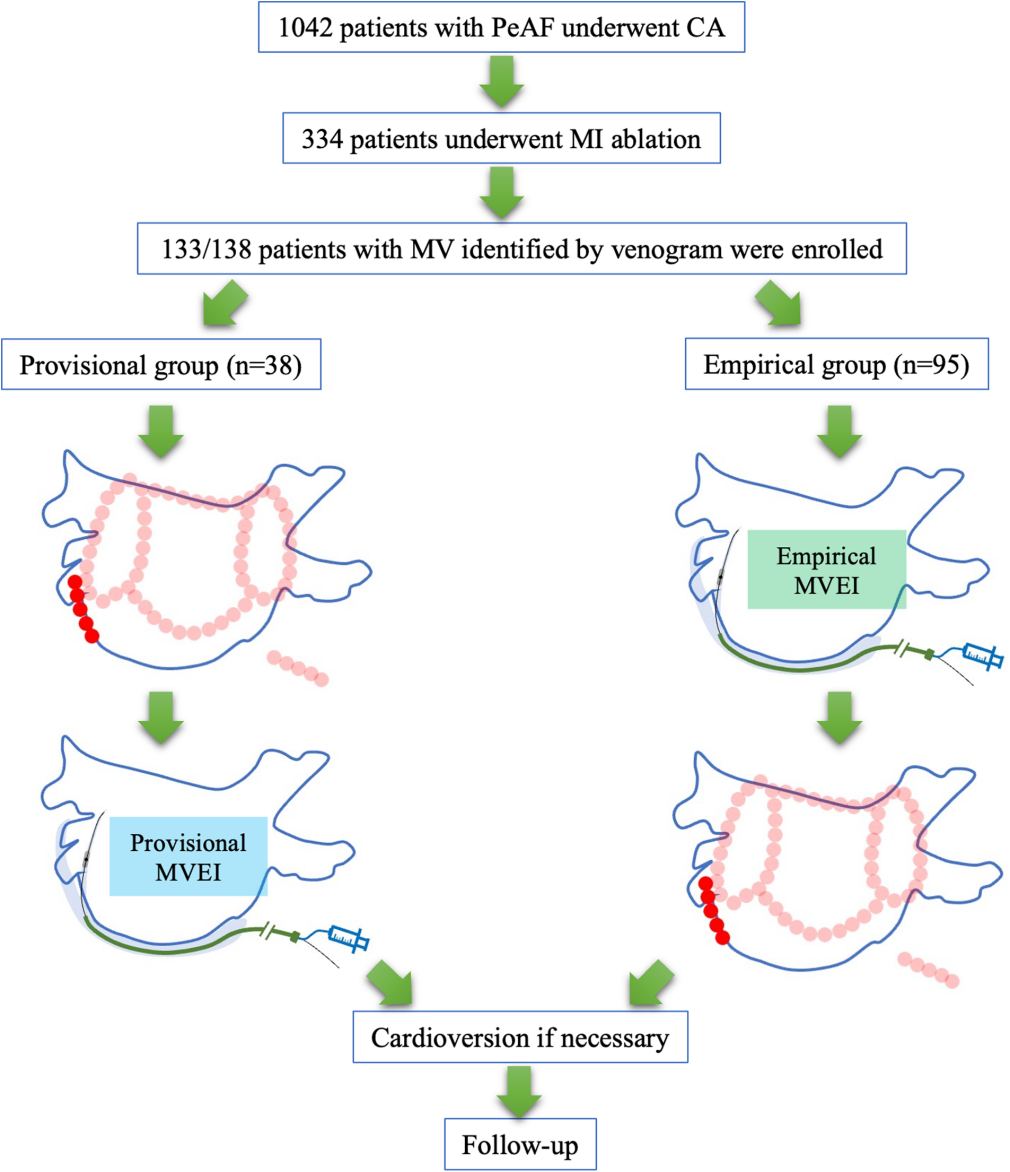
Study flowchart. PeAF, persistent atrial fibrillation; CA, catheter ablation; MI, mitral isthmus; MVEI, Marshall vein ethanol infusion.

Among all consecutive patients with PeAF who underwent CA during the period between April 2019 and March 2022 in our center, those who underwent CS venogram and first-time AF ablation were included retrospectively. Patients who had MV and complete data were enrolled in either the provisional group (MVEI was performed when conventional endocardial and/or epicardial ablation procedures were inadequate to achieve bidirectional MI block) or in the empirical group [MVEI was performed empirically before MI CA (Figure 1]. All patients met the indications of CA on the basis of the current guidelines (10, 11). The exclusion criteria were as follows: (i) permanent AF refractory to medical or electrical cardioversion; (ii) associated structural heart disease other than left ventricular hypertrophy; (iii) a previous AF CA procedure or cardiac surgery; and (iv) AF with valvular disease ⩾grade 2. All patients were provided written informed consent. All antiarrhythmic drugs were discontinued for at least 5 half-lives and amiodarone was stopped at least 4 weeks before the ablation procedure. The study protocol adhered to the principle of the Declaration of Helsinki and was approved by the local Institutional Review Board (register number 2022RS105).

### 2.2. Ablation procedures

The absence of thrombus in the LA or left atrial appendage (LAA) was confirmed by transesophageal echocardiography within 48 hrs before the procedure. All ablation procedures were performed under local anesthesia and deep sedation. Intravenous heparin was administered after femoral venous access to maintain an intraprocedural activated clotting time of 250–350 s. Intracardiac electrograms were recorded using the multichannel electrophysiology system (EP-Workmate, Abbott, USA), and radiofrequency CAs were performed under the guidance of 3D electroanatomic mapping systems (Carto3, Biosense-Webster, USA or Ensite Precision, Abbott, USA). A decapolar mapping CS catheter was introduced via the left femoral vein and double transseptal punctures were performed under the guidance of fluoroscopy. A geometrical reconstruction of the LA and PVs and activation/voltage mapping were done using multipolar mapping catheters (Pentaray Nav, Biosense-Webster, USA or Doubleloop, Abbott, USA). An open irrigated-tip contact force (CF)-sensing catheter (Thermocool SmartTouch, Biosense-Webster, USA or TCQ, Abbott, USA) was applied to deliver radiofrequency ablations. Circular or linear ablation lesions were delivered at a quantitative ablation target [for the LA anterior wall, LA roof, MI endocardial aspect, and cavotricuspid isthmus (CTI): an ablation index (AI) of 500–550 and a lesion size index (LSI) of 5.0–5.5; for the LA posterior wall, LA bottom, and MI epicardial aspect within the CS: an AI of 350–400 and an LSI of 3.5–4.0] and at a center-to-center interlesion distance <6 mm with a target CF of 5–20 g (12).

MVEI has been described in detail in previous studies (4, 6, 8, 9, 13–15). Briefly, the presence and location of the MV were identified by using a subselective venogram with a 6-Fr JR 4.0 guiding catheter via a steerable sheath (Agilis NxT, Abbott, USA or Vizigo, Biosense-Webster, USA) cannulated into the CS. Then, a 0.014″ angioplasty guidewire (Runthrough, Terumo, Japan) was introduced into the MV, over which an over-the-wire balloon (Emerge, Boston Scientific, USA) was advanced to the ostium of the MV. A selective venogram was performed to delineate the branching pattern of the MV and to identify the occlusion of the MV by inflating the balloon. Then, two infusions of 6–8 ml 98% ethanol were injected gently into the occluded MV at its distal and proximal part, respectively. Repeated contrast injection was administered to check the MV after ethanol infusions.

For patients in the provisional group, bilateral pulmonary vein isolation (PVI) was performed initially. Spontaneous or inducible MI-dependent atrial tachycardia (AT) was confirmed by activation and entrainment mapping. Endocardial MI linear ablation and/or epicardial ablation within the CS at the reciprocal aspect to the MI line were applied. If the RF ablation was insufficient to achieve the bidirectional block of MI, MVEI was performed. Additional linear ablations such as LA roof line, posterior BOX line (roof plus posterior linear ablations with the isolation of the posterior wall as the endpoint), CTI line, anterior wall line, or superior vena cava (SVC) isolation were performed at the discretion of the surgeon (Figure 1).

For patients in the empirical group, MVEI was performed before RF ablation procedures. Voltage mapping before and after MVEI were performed and compared. Then, bilateral PVI and linear ablations including the MI line from the endocardial aspect were delivered (Figure 1).

Sinus rhythm was restored either by CA of tachyarrhythmias, intravenous injection of ibutilide, and MVEI or by synchronous electrical cardioversion (SECV).

The endpoint of the ablation procedure was the bidirectional block between all PVs and LA and across all ablation lines. A Complete bidirectional block across the MI line was confirmed by the activation sequence in the CS showing a proximal-to-distal pattern when pacing from the LAA. High-density activation mapping was performed to locate the conducting gap when the block was incomplete and additional endocardial and/or epicardial ablation applications were delivered at gaps to achieve complete block.

### 2.3. Postprocedure treatments and follow-up

All antiarrhythmic drugs were discontinued in all patients after the 3-month blanking period. Long-term oral anticoagulants were recommended on the basis of thromboembolic risk evaluations in accordance with the current guidelines (11, 16). Clinic outpatient visits such as echocardiography and 24-h Holter tests were arranged regularly at 3, 6, and 12 months after the index ablation procedure and then every 6 months or whenever patients experienced symptoms. Clinical recurrence was defined as any documented episode of AF or ATs (including atrial flutter and tachycardia) lasting for at least 30 s after the blanking period (11, 16).

### 2.4. Statistical analysis

Statistical analysis was performed using SPSS software (version 26.0, IBM, USA). Continuous variables were expressed as mean ± standard deviation (SD) or median (interquartile range, IQR) for non-normal distributions, and categorical variables were reported as frequency (percentage). Parametric (Student's *t*-test) or nonparametric tests (Mann–Whitney *U* test and chi-square test/Fisher's exact test) were used to compare differences in clinical and ablation parameters between the two groups. Kaplan–Meier analyses with log-rank tests were used to calculate AF/AT recurrence-free survival over time and to compare recurrence rates between the two groups. Univariate and multivariate Cox regression analyses were used to evaluate the predictors of atrial arrhythmia recurrence. A *P*-value of <0.05 (two-sided) was considered statistically significant.

## 3. Results

### 3.1. Baseline characteristics

A total of 334 patients underwent MI ablation from 1,042 consecutive PeAF patients who were screened. One hundred and thirty-eight patients underwent CS venogram and the absence of MV was identified in 5 (3.6%) patients. The remaining 133 patients were divided into either the provisional group (*n* = 38) or the empirical group (*n* = 95, Figure 1). The baseline characteristics of all participants were comparable between the two groups (Table 1).

**Table 1:** Comparison of baseline characteristics.

Variable	Provisional group (*n* = 38)	Empirical group (*n* = 95)	*P-*values
Female, *n* (%)	17 (44.7)	31 (32.6)	0.231
Age, mean ± SD (years)	62.3 ± 11.1	65.0 ± 8.4	0.133
History of AF, median (IQR) (months)	48.0 (18.0, 90.0)	12.0 (3.0, 48.0)	0.139
BMI, mean ± SD (kg/m^2^)	25.7 ± 3.7	24.9 ± 3.0	0.227
Hypertension, *n* (%)	19 (50.0)	50 (52.6)	0.849
Diabetes mellitus, *n* (%)	6 (15.8)	13 (13.7)	0.787
Renal dysfunction, *n* (%)	2 (5.3)	5 (5.4)	1.000
Previous CVA, *n* (%)	4 (10.5)	12 (12.6)	0.966
Previous bleeding, *n* (%)	0 (0)	5 (5.4)	0.321
LA diameter, mean ± SD (mm)	42.8 ± 5.2	44.3 ± 4.6	0.119
LVEF, mean ± SD (%)	62.6 ± 6.0	61.7 ± 8.7	0.574
CHA_2_DS_2_-VASc score, median (IQR)	2.0 (0.5, 4.0)	1.0 (2.0, 4.0)	0.605
HAS-BLED score, median (IQR)	1.0 (0, 2.0)	1.0 (1.0, 2.0)	0.261
Oral anticoagulants, *n* (%)			
Rivaroxaban	29 (76.3)	78 (82.1)	0.473
Dabigatran	8 (21.1)	15 (15.8)	0.612
Warfarin	1 (2.6)	2 (2.1)	1.000

SD, standard deviation; AF, atrial fibrillation; IQR, interquartile range; BMI, body mass index; CVA, cerebral vascular accident, including stroke and transient ischemic attack; LA, left atrium; LVEF, left ventricular ejection fraction.

### 3.2. Procedural characteristics

As seen in Table 2, more spontaneous or inducible ATs were recorded in the provisional group of patients (57.9% vs. 7.4%, *P *< 0.001), in which more ATs were MI-dependent (50.0% vs. 2.1%, *P *< 0.001). Endocardial contiguous linear lesions between the left inferior pulmonary vein (LIPV) and the mitral annulus (MA) were applied in all patients, while more epicardial ablation lesions within the CS were delivered in the provisional group of patients (26.3% vs. 9.5%, *P *= 0.016). The distribution of other ablation strategies was comparable between the two groups. A higher incidence of SR restoration by CA was observed in the provisional group (86.8% vs. 11.7%, *P < *0.001), while more arrhythmia terminations were achieved by SECV in the empirical group (13.2% vs. 78.9%, *P *< 0.001).

**Table 2:** Comparison of procedure-related results.

	Provisional group (*n* = 38)	Empirical group (*n* = 95)	*P*-Value
Mapping system Carto3/Ensite precision, *n* (%)	30 (78.9)/8 (21.1)	85 (89.5)/10 (10.5)	0.158
Spontaneous/induced AT, *n* (%)	22 (57.9)	7 (7.4)	<0.001
MI-dependent AT	19 (50.0)	2 (2.1)	<0.001
Ablation strategies, *n* (%)			
PVI plus MI_endo_ line	38 (100)	95 (100)	–
MI_epi_ ablation	10 (26.3)	9 (9.5)	0.016
MVEI	21 (55.3)	95 (100)	<0.001
LA roof line	31 (81.6)	75 (78.9)	0.815
BOX line	6 (16.2)	18 (19.4)	0.805
Anterior wall line	0 (0)	4 (4.2)	0.326
CTI line	8 (21.6)	19 (20.2)	1.000
CFAE elimination	3 (7.9)	9 (9.5)	1.000
SVC isolation	7 (18.9)	11 (11.6)	0.397
Arrhythmia termination patterns, *n* (%)			
Catheter ablation	33 (86.8)	11 (11.7)	<0.001
Ibutilide IV	0 (0)	6 (6.3)	0.182
MVEI	0 (0)	3 (3.2)	0.557
SECV	5 (13.2)	75 (78.9)	<0.001
Ablation-related parameters			
Procedure time, median (IQR) (min)	120.0 (112.5, 156.0)	120.0 (103.5, 138.5)	0.255
Total fluoroscopy time, median (IQR) (min)	6.4 (4.2, 9.3)	9.5 (5.9, 11.6)	0.019
Total fluoroscopy exposure, median (IQR) (mGy)	69.0 (25.3, 160.2)	122.0 (62.5, 234.1)	0.010
Total ablation duration, mean ± SD (s)	2,068.1 ± 877.2	2,238.4 ± 738.8	0.307
MI_endo_ ablation time, mean ± SD (s)	455.9 ± 192.2	368.0 ± 163.6	0.038
Ethanol infusion, mean ± SD (ml)	8.5 ± 3.4	9.4 ± 3.0	0.165
Procedure-related complications, *n* (%)			
Vascular access complications	1 (2.6)	3 (3.2)	1.000
Pericardial effusion	0 (0)	1 (1.1)	1.000

AT, atrial tachycardia; MI, mitral isthmus; PVI, pulmonary vein isolation; MI_endo_, endocardial aspect of the mitral isthmus; MI_epi_, the epicardial aspect of the mitral isthmus, mainly localized in the distal coronary sinus and great cardiac vein; MVEI, Marshall vein ethanol infusion; CTI, cavotricuspid isthmus; CFAE, complex fractionated atrial electrograms; SVC, superior vena cava; IV, intravenous injection; SECV, synchronous electrical cardioversion; other abbreviations as in Table 1.

The total procedure time did not differ between the two groups. However, more fluoroscopy time (6.4[4.2, 9.3] vs. 9.5[5.9, 11.6] min, *P *= 0.019) and radiation exposure (69.0[25.3, 160.2] vs. 122.0[62.5, 234.1] mGy, *P *= 0.010) were recorded in the empirical group. Although the total ablation time was similar between the groups, the endocardial ablation time in the empirical group was significantly less (455.9 ± 192.2 vs. 368.0 ± 163.6 s, *P *= 0.038).

The incidences of procedure-related complications were low and similar between the groups (Table 2).

### 3.3. Long-term follow-up and predictors of arrhythmia recurrence

**Figure 2:**
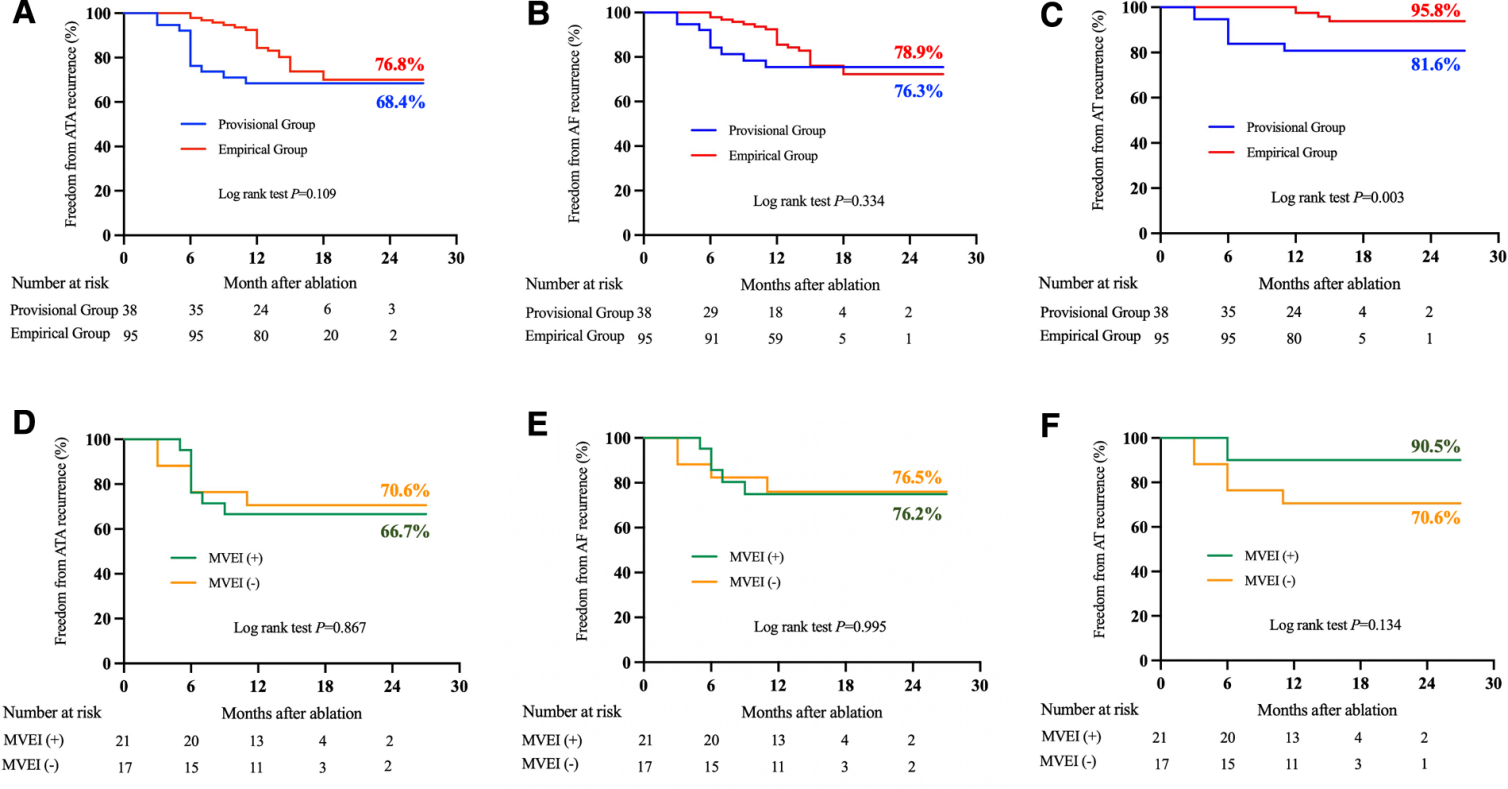
Comparison of atrial tachyarrhythmias recurrences between two groups (panels A–C) and among patients with or without MVEI in the provisional group (panels D–F) during follow-up. ATA, atrial tachyarrhythmia; AF, atrial fibrillation; AT, atrial tachycardia; MVEI, Marshall vein ethanol infusion.

After a 16.5 ± 4.4-month follow-up, the AT-free success rate was significantly higher in the empirical group of patients (95.8% vs. 81.6%, log-rank *P *= 0.003, Figure 2C). However, freedom from atrial tachyarrhythmias (ATA, combining AF and AT, 76.8% vs. 68.4%, log-rank *P *= 0.109, Figure 2A) and AF (78.9% vs. 76.3%, log-rank *P *= 0.334, Figure 2B) recurrence between the groups was comparable.

When compared with those patients who underwent MVEI in the provisional group, similar success rates of freedom from ATA (70.6% for MVEI[−] vs. 66.7% for MVEI[+], log-rank *P *= 0.867), AF (76.5% for MVEI[−] vs. 76.2% for MVEI[+], log-rank *P *= 0.995), or AT (70.6% for MVEI[−] vs. 90.5% for MVEI[+], log-rank *P *= 0.134) were observed in patients without MVEI (Figures 2D–F).

In the univariate Cox regression analysis, the LA diameter, history of hypertension, performance of empirical MVEI, intraprocedural AT, especially MI-dependent AT, and restoration of SR by ablation were associated with lower AT recurrence (all *P* values <0.05). However, the performance of empirical MVEI had an independent association with lower AT recurrence (*P *= 0.037, Table 3) in the multivariate COX regression analysis.

**Table 3:** Univariate and multivariate cox regression analyses of AT recurrence.

	Univariate analysis		Multivariate analysis	
	HR (95% CI)	*P*-value	HR (95% CI)	*P*-value
Age	0.98 (0.92–1.04)	0.471	0.99 (0.93–1.05)	0.710
LA diameter (mm)	0.90 (0.80–1.00)	**0**.**040**	0.92 (0.82–1.03)	0.127
BMI (kg/m^2^)	1.02 (0.85–1.22)	0.859		
Hypertension	0.19 (0.04–0.88)	**0**.**034**		
Empirical MVEI	0.19 (0.05–0.64)	**0**.**008**	0.25 (0.07–092)	**0**.**037**
Ethanol volume (ml)	0.88 (0.74–1.04)	0.140		
Intraprocedural AT	3.59 (1.09–11.82)	**0**.**036**		
MI-dependent AT	3.86 (1.12–13.28)	**0**.**032**		
Roof line ablation	2.43 (0.31–19.06)	0.397	1.96 (0.24–15.84)	0.527
BOX isolation	0.50 (0.06–3.94)	0.510	0.61 (0.08–5.00)	0.645
CTI line ablation	1.42 (0.37–5.52)	0.610	1.13 (0.28–4.66)	0.862
SVC isolation	0.67 (0.09–5.26)	0.705		
MI epicardial ablation	0.62 (0.08–4.85)	0.649		
Ablation to SR	5.90 (1.56–22.29)	**0**.**009**		
Procedure time (min)	1.00 (0.99–1.02)	0.646		
Ablation time (min)	1.00 (1.00–1.00)	0.663		

CI, confidence interval; SR, sinus rhythm; other abbreviations as in Tables 1, 2.

Bold values are of statistic significance.

## 4. Discussion

The present study evaluated the efficacy and safety of empirical MVEI in PeAF patients undergoing the index MI ablation procedure. The main findings are as follows. (1) Less spontaneous or inducible ATs were documented when MVEI was performed empirically before the conventional ablation procedure. (2) More epicardial ablation procedures were required to achieve a bidirectional block of MI if MVEI was performed in a provisional manner. (3) Although more fluoroscopy exposures were recorded, performing MVEI empirically helped reduce endocardial MI ablation time. (4) Empirical MVEI was independently associated with lower long-term AT-free success.

### 4.1. Electrophysiological considerations of MV

In clinical scenarios that include documented MI-dependent macro reentry ATs or anatomical lesion sets that aim at atrial compartmentalization beyond PVI, endocardial and/or epicardial linear ablation connecting LIPV and MA would be performed. However, to achieve a bidirectional block across MI might be challenging sometimes for the occurrence of unavoidable residual conduction gaps (17). A non-transmural lesion set across MI is one of the main risk factors for gap conduction and tachyarrhythmia recurrence (3).

The MV surrounded by the Marshall bundle (MB) descending obliquely over the epicardial aspect of the LA lateral ridge has been considered one of the non-PV origins of ectopy initiating atrial tachyarrhythmias (17, 18). A distinct MB potential during SR and rapid and fractionated activations during AF could be recorded with a thin multielectrode catheter inserted within the MV (2). Ectopic activities from the distal or middle part of the MV triggering AF could be documented and eliminated to achieve MI block (19, 20). MB-dependent ATs such as macro- and localized reentries could be visualized by high-density activation mapping and confirmed with entrainment along the circuits. Although all of those reentries could be terminated by RF ablation and complementary MVEI, higher recurrence was observed after RF ablation compared with ethanol infusion (21). In our study, the incidence of intraprocedure spontaneous or inducible ATs, especially MI-dependent ones, was significantly lower in the empirical group than in the provisional group. It is hypothesized that ethanol infusion prior to RF ablation might modify the substrate of MI-dependent tachyarrhythmias partially. Whether empirical MVEI can reduce MI-dependent arrhythmogenicity has yet to be proved with prospective studies.

### 4.2. The role of MVEI played in PeAF ablation

Retrograde ethanol infusion into the MV has been recognized as a therapeutic tool in AF ablation (4, 13–15, 20, 22). Adjunctive MVEI can facilitate the efficacy of PVI (14, 23). Anatomical ablation strategies such as MB elimination, PVI, and line completion can also improve the success rate in patients with PeAF (6–8, 24). In patients with non-paroxysmal AF, added MVEI can reduce the risk of atrial arrhythmia recurrence and improve the procedural termination rate significantly compared with PVI or PVI plus substrate modification. Multivariate analysis revealed MEVI as an independent predictor of freedom from AF and AT recurrence (24). In the prospective, single-center Marshall-PLAN study, the full lesion set (PVI plus MVEI and linear lesions across MI, roof, and CTI) was completed successfully in 91% patients. The one-year rate of freedom from AF/AT after a single procedure was 72% in the overall cohort and 79% in the complete lesion set subgroup (8). The “conventional 2C3l” approach comprising bilateral PVI and bidirectional block in the roofline, CTI, and MI can also be facilitated with an upgraded strategy of additional MVEI with a shorter LPV antrum and MI ablation time and lower recurrence rate (7). The VENUS randomized multicenter trial also demonstrated remarkably higher rates of freedom from AF/AT after a single procedure in PeAF patients who underwent CA plus MVEI. The rates of reduction of AF burden, freedom from AF after multiple procedures, and success in achieving perimetral block also improved significantly in MV-treated patients (6). In our study, long-term success rates from AF recurrence in both groups (76.2% and 78.9%, respectively) could also be documented when MVEI was performed. The durability of the MVEI-related atrial lesion could be identified by comparing the area of the acute low bipolar voltage region in the index procedure and that of the chronic scar in the redo procedure (25). However, all procedures in this study were performed by using SmartTouch or TCQ ablation catheters. We do believe that the rate of achievement of the endocardial MI block would be improved and outcomes with or without MVEI would be reduced with a more wide use of new-generation ablation catheters (e.g., SmartTouch SurroundFlow, Biosense-Webster, USA).

### 4.3. Should MVEI be the first-step ablation strategy?

Whether MVEI should be the first step in the PeAF ablation procedures has seldom been investigated. A secondary analysis of the VENUS trial indicated that adding MVEI to CA favored the outcomes of PeAF patients with perimetral block in high-volume centers (26). However, the VENUS trial did not indicate whether MVEI should be performed empirically prior to CA. Recently, Gillis et al. investigated the added value of MVEI as a first step in CA-guided MI block in a small-volume randomized study (27). Added MVEI could be associated with a higher incidence of MI block after endocardial and epicardial ablation procedures and reduced number of ablations needed to achieve MI block. In our study, the need for performing epicardial ablation procedures to achieve MI block (26.3% vs. 9.5%, *P *= 0.016) and reduce endocardial MI ablation time (455.9 ± 192.2 vs. 368.0 ± 163.6 min, *P *= 0.038) was markedly lower in the empirical group of patients than in the provisional group of patients.

Moreover, the efficacy of MVEI might also be reduced because of the complexity of the MV anatomy (14). This could partially explain why in our study, the AF-free success rate did not show statistical improvement after the implementation of the empirical MVEI strategy (78.9% vs. 7 6.3%, log-rank *P *= 0.334). However, ultrahigh-resolution mapping and entrainment pacing along the MA demonstrated that about two-thirds of the perimitral ATs were MV-dependent (28). Addition of MVEI to CA could reduce the incidence of AT recurrence when patients underwent empirical ethanol infusion (95.8% vs. 81.6%, log-rank *P *= 0.003).

## 5. Limitations

Firstly, we had not achieved the encouraging results like those from the VENUS study in improvement of freedom from AF either in the provisional group or the empirical group limited to the volume of this retrospective study. However, a reduction of long-term AT incidence was observed in the empirical group. A future prospective multicenter randomized study with a large number of participants is needed to validate the conclusions of the present study.

Secondly, to compare the efficacy of the empirical MVEI, we preclude the cases without the Marshall vein. This selection bias might overestimate the advantage of MVEI in reducing postprocedure recurrence from AF and/or AT.

Finally, since MVEI was not performed in all patients in this study, the areas of the low-voltage zone before and after the performance of MVEI were not investigated quantificationally. Therefore, measurements and comparisons can be applied in future studies.

## 6. Conclusion

Empirical Marshall vein ethanol infusion performed before conventional catheter ablation could significantly reduce endocardial mitral isthmus ablation time and risk of atrial tachycardia recurrence after a long-term follow-up in patients with persistent atrial fibrillation who underwent index mitral isthmus ablations. However, large-scale prospective studies are warranted to verify our findings.

## Data Availability

The original contributions presented in the study are included in the article/**Supplementary Material**, and further inquiries can be directed to the corresponding author.
